# Age-Dependent Changes in Calcium Regulation after Myocardial Ischemia–Reperfusion Injury

**DOI:** 10.3390/biomedicines11041193

**Published:** 2023-04-17

**Authors:** Maria Bencurova, Terezia Lysikova, Katarina Leskova Majdova, Peter Kaplan, Peter Racay, Jan Lehotsky, Zuzana Tatarkova

**Affiliations:** Department of Medical Biochemistry, Jessenius Faculty of Medicine in Martin, Comenius University in Bratislava, 03601 Martin, Slovakia

**Keywords:** aging, heart, ischemia, reperfusion, Ca^2+^-ATPase, calcium handling

## Abstract

During aging, heart structure and function gradually deteriorate, which subsequently increases susceptibility to ischemia–reperfusion (IR). Maintenance of Ca^2+^ homeostasis is critical for cardiac contractility. We used Langendorff’s model to monitor the susceptibility of aging (6-, 15-, and 24-month-old) hearts to IR, with a specific focus on Ca^2+^-handling proteins. IR, but not aging itself, triggered left ventricular changes when the maximum rate of pressure development decreased in 24-month-olds, and the maximum rate of relaxation was most affected in 6-month-old hearts. Aging caused a deprivation of Ca^2+^-ATPase (SERCA2a), Na^+^/Ca^2+^ exchanger, mitochondrial Ca^2+^ uniporter, and ryanodine receptor contents. IR-induced damage to ryanodine receptor stimulates Ca^2+^ leakage in 6-month-old hearts and elevated phospholamban (PLN)-to-SERCA2a ratio can slow down Ca^2+^ reuptake seen at 2–5 μM Ca^2+^. Total and monomeric PLN mirrored the response of overexpressed SERCA2a after IR in 24-month-old hearts, resulting in stable Ca^2+^-ATPase activity. Upregulated PLN accelerated inhibition of Ca^2+^-ATPase activity at low free Ca^2+^ in 15-month-old after IR, and reduced SERCA2a content subsequently impairs the Ca^2+^-sequestering capacity. In conclusion, our study suggests that aging is associated with a significant decrease in the abundance and function of Ca^2+^-handling proteins. However, the IR-induced damage was not increased during aging.

## 1. Introduction

Cardiovascular diseases are the leading cause of death worldwide, taking more than 17.9 million lives each year (one-third of all deaths worldwide), with more than four out of five deaths due to heart attack or stroke [1]. Despite reduced mortality due to important advances in the treatment of myocardial infarction (therapeutic targets, peptides, drugs), there is a need to limit the extent of infarct size after reperfusion injury, thus preventing the onset and severity of heart failure [2]. The role of age in tolerance to ischemic injury has been studied for several decades, but the results are controversial [3,4,5,6]. Some studies suggest that tolerance to ischemia–reperfusion injury, and its prevention by preconditioning, decreases with age [5,6,7,8], while other studies show that tolerance to ischemia or ischemic preconditioning is not changed during aging [3]. Aging elevates the susceptibility of the heart to ischemia and myocardial infarction [9], with the highest occurrence (over 50%) of ischemic heart diseases in people older than 70 [10]. Moreover, heart contractility recovery is also attenuated by age, thus influencing the therapeutic effect and intervention time point, which differ between young and elderly patients.

To ensure the normal physiological function of the heart, a sufficient amount of energy is required, the production of which is mainly provided by mitochondria [11]. The pathophysiology of cardiac cells during aging is a culmination of altered response to adrenergic stimulation [12] and changes in partially immobile mitochondria [13], such as structural deformation (reorientation and loss of cristae, swelling) [14,15], mitochondrial fragmentation or reduced biogenesis [16,17], increased reactive oxygen species (ROS) production [18], altered intracellular homeostasis of Ca^2+^ [19,20], and decreased electron transport chain activity [21]. Dysfunctional mitochondria and organelles such as sarco/endoplasmic reticulum (SR/ER) accumulation often lead to abnormal function of cardiomyocytes or vascular endothelial cells [11]. ER as a multifunctional organelle is implicated in protein synthesis and folding, lipid biosynthesis, and participates in peroxisome formation [22]. However, its dysfunction promotes the development of proteotoxic ER stress [23]. While altered proteostasis is considered an important feature of the aging heart, reduced protein synthesis and turnover are associated with decreased efficiency of autophagy and the ubiquitin–proteasome system [24].

The unequivocal fact is that Ca^2+^ plays a critical role in cardiac cells, coupling electrical excitation to mechanical contraction (excitation–contraction coupling, ECC) with each heartbeat [25]. The sarcoplasmic reticulum is a high-capacity reservoir of intracellular Ca^2+^ required for the regulation of muscle activity [26,27] that is mediated by the cooperation of two essential Ca^2+^-release channels, inositol 1,4,5-trisphosphate receptors (IP3Rs) and ryanodine receptors (RyRs) [28], and by the activity of SR/ER Ca^2+^-ATPase (SERCA2a), which is responsible for the reuptake of intracellular Ca^2+^ into SR, thus achieving ECC [29]. Activated IP3R is a Ca^2+^-induced Ca^2+^ release channel similar to RyR, since both require low Ca^2+^ levels for activation and at high concentrations are inactivated [30]. However, they possess distinct physiological and pharmacological profiles [28]. While RyR is the primary Ca^2+^ channel responsible for generating the cell-wide Ca^2+^ transients during ECC in the heart, the release of Ca^2+^ via IP3Rs can elicit ECC-modulating effects [25], depending on its location. Aging attenuates Ca^2+^ transients, which are significantly prolonged [31] due to reduced SERCA2a expression and ROS-induced over-activation of RyRs [32]. Dysfunctional Ca^2+^-ATPase [23,33] causes delayed re-uptake of Ca^2+^ by SR, resulting in Ca^2+^ transient impairment [34], but interestingly the expression of SERCA2a has been reported to be different in each part of the heart during aging [35]. It may be a protective effort of the aging heart to maintain optimal activity, but the results are controversial yet.

The proximity between SR and mitochondria allows for the regulation of a wide spectrum of processes [36]. Ca^2+^ homeostasis is controlled by the protein complex that consists of IP3R/RyR and mitochondrial voltage-dependent anion channel 1, both stabilized by glucose-regulated protein 75 [37]. The inner mitochondrial membrane Ca^2+^ uniporter mediates Ca^2+^ uptake and subsequently activates the synthesis of ATP. In response to mitochondrial ATP, the rapid release of Ca^2+^ from the sarcoplasmic reticulum into the cytoplasm ensures contraction [38]. Relaxation ensues when Ca^2+^ is extruded from the cytosol via the Na^+^/Ca^2+^ exchanger and sequestered into the sarcoplasmic reticulum [39]. However, any perturbations in intracellular Ca^2+^ handling can alter the contractility and electrical stability of the heart. A brief period of ischemia, which is not associated with irreversible myocardial damage but results in altered cardiac contractility, is known as myocardial stunning [40]. There are several hypotheses described and recently summarized [41], however, the mechanisms are not completely understood.

The objective of our study was to investigate the role of aging in tolerance to ischemia–reperfusion injury. We also aimed to evaluate the contribution of intracellular Ca^2+^ homeostasis dysregulation in these pathological conditions.

## 2. Materials and Methods

### 2.1. Animals

Male Wistar rats (n = 45) supplied by the Institute of Experimental Pharmacology, Slovak Academy of Sciences (Dobra Voda, Slovakia) were divided into three groups according to age, namely, 6-month-old (n = 15), 15-month-old (n = 15), and 24-month-old (n = 15) rats, with average weight 398 ± 11 g, 459 ± 33 g, and 468 ± 63 g, respectively. Animals were maintained as we described previously [42] in a temperature-controlled room at 22 ± 2 °C on a 12-h light/dark cycle, with the light on at 6 a.m., and with access to food and water *ad libitum*.

**Figure 1:**
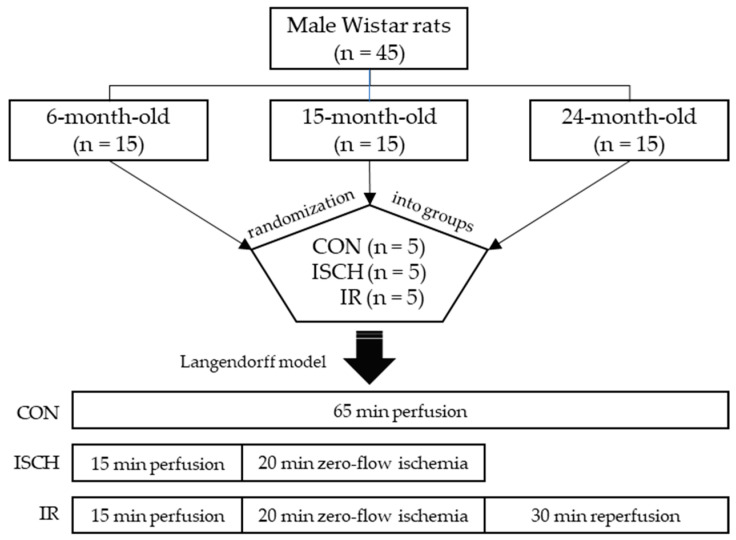
Schematic representation of the experimental protocol. A model of myocardial ischemia and reperfusion using rats of three age groups and subdivided into control group (CON), ischemic group (ISCH), and ischemia-reperfusion group (IR).

All experiments were performed following the EU directive (2010/63/EU) concerning the protection of animals used for scientific purposes and approved by the Ethical Committee of Jessenius Faculty of Medicine in Martin (No. 2010/04/15) and by the State Veterinary and Food Department of Slovakia (No. 554/11-221/3, 16.12.2016). Animals of individual age groups were randomly chosen and subjected to various experimental conditions (Figure 1).

Our study used the Langendorff model of a 20-min ischemic insult on perfused rat hearts, since there is evidence that this period of ischemia should be associated with minimal necrosis and the ability of hearts to reverse post-ischemic injury completely [43].

### 2.2. Langendorff Model of Ischemia–Reperfusion

The animals of the 3 age groups were decapitated after anesthetization by 3% halothane in O_2_/NO mixture (1:2). After rapid excision, the hearts were placed in an ice-cold Krebs–Henseleit (K–H) solution (pH 7.4) that contained NaCl (135.0 mM), KCl (5.4 mM), MgCl_2_ (0.9 mM), NaHCO_3_ (24.0 mM), NaH_2_PO_4_ (1.2 mM), and CaCl_2_ (1.8 mM) with glucose (10.0 mM). K–H solution was continuously saturated with 95% O_2_ and 5% CO_2_. Excised hearts were immediately cannulated through the aorta to ensure spontaneous sinus rhythm and perfused with the K–H solution according to the Langendorff method using the Langendorff system (ML870B2, ADInstruments, Spechbach, Germany) at 37 °C and a constant pressure of 73 mmHg. Isovolumetric left ventricular pressure was measured with a latex balloon coupled to a pressure transducer. A balloon was inserted into the left ventricle through the left atrium. Its volume was adjusted to maintain an initial left ventricular end-diastolic pressure at 8–12 mmHg.

A PowerLab 8/30 Data Acquisition System with LabChart Software (ADInstruments, Spechbach, Germany) was used for continuous recording and processing of hemodynamic parameters and temperature. In the end, all hearts were frozen and stored at −80 °C for later use.

### 2.3. Preparation of Tissue Homogenates and SR Vesicles

Tissue homogenates were prepared from rat hearts of the 3 age groups, each consisting of CON, ISCH, and IR subgroups. Frozen tissues were thawed in ice-cold homogenization buffer (1:10) containing 30.0 mM KH_2_PO_4_, 0.3 M sucrose, 5.0 mM EDTA (pH 7.0), and before use protease inhibitor 0.3 mM phenylmethylsulfonyl fluoride was added. All hearts underwent homogenization at 1000 rpm 5× in 25/20 s intervals using precooled Teflon pestle in Potter–Elvehjem homogenizer. Next, cardiac sarcoplasmic reticulum vesicles were isolated from homogenates in several steps by differential centrifugation as described previously [44]. Briefly, homogenates were centrifuged at 4000× *g* for 15 min, filtered through gauze, and centrifuged again (14,000× *g*, 15 min). In the next step, the Optima L-100 XP ultracentrifuge (Beckman Coulter, Brea, CA, USA) was used to separate the supernatant at 120,000× *g* for 70 min. The pellet was resuspended in a solution containing 10.0 mM imidazole and 650.0 mM KCl (pH 6.8), incubated for 30 min on ice, and centrifuged again at 4400× *g* for 10 min. Finally, obtained supernatant was centrifuged for 70 min at 20,000× *g* and the resulting pellet was resuspended in an isolation solution (30.0 mM imidazole, 60.0 mM KCl, 2.0 mM MgCl_2_; pH 7.0). All steps for isolation were performed at 4 °C. Isolated SR fractions were used to determine SR/ER Ca^2+^-ATPase activity.

The commercially available DC Protein assay kit (500-0111, Bio-Rad Laboratories, Hercules, CA, USA) was used to measure total protein concentration in all isolated samples. The measurement was performed according to the manufacturer’s instructions using a BioTek Synergy H4 hybrid microplate reader (Agilent Technologies, Santa Clara, CA, USA).

### 2.4. Assay of Ca^2+^-ATPase Activity

Cardiac sarco-endoplasmic reticulum Ca^2+^-ATPase activity was determined according to the protocol [44] on the PharmaSpec UV-1700 spectrophotometer (Shimadzu, Japan). Shortly, Mg^2+^-dependent Ca^2+^-stimulated activity was measured based on the rate of inorganic phosphate release. The reaction medium contained HEPES (20.0 mM, pH 7.0), KCl (100.0 mM), MgCl_2_ (5.0 mM), NaN_3_ (5.0 mM), ATP (5.0 mM), EGTA (0.1 mM), Ca^2+^ ionophore A23187 (5.0 μg/mL), and various amounts of 1.0 mM CaCl_2_ and 0.05 mg/mL SR proteins. The reaction started with the addition of ATP and was terminated after a 15-min incubation at 37 °C by adding 10% trichloroacetic acid to the reaction mixture and cooling the samples on ice. Kinetic parameters V_max_ and K_Ca_ were calculated by the nonlinear least-squares method as V_max_/V = 1 + (K_Ca_/[Ca^2+^]), where V is the velocity, V_max_ is the maximum Ca^2+^-ATPase activity, and K_Ca_ is free calcium concentration [Ca^2+^] at V_max_/2.

### 2.5. Dot Blot

Dot blot analysis was used to visualize 314 kDa inositol-1,4,5-triphosphate receptor and 560 kDa ryanodine receptor. Homogenates were diluted to 4.0 µg/µL and 2.5 µL drops (10.0 µg of protein) were spotted onto a 0.45 µm nitrocellulose membrane (1620115; Bio-Rad Laboratories, Hercules, CA, USA). After drying, primary and secondary antibodies were applied to membranes according to the immunodetection procedure described below.

### 2.6. Western Blot and Immunodetection

Proteins (40 µg per sample) from tissue homogenates were separated using sodium dodecyl sulfate–polyacrylamide gel electrophoresis with 8%, 10%, or 15% gels (based on Mw of target protein) under denaturing conditions (3 min at 95 °C). Separated proteins underwent a semi-dry transfer at 60 mA per gel onto nitrocellulose membranes using Trans-Blot^®^ SD Semi-Dry Transfer Cell apparatus (1620115; Bio-Rad Laboratories, Hercules, CA, USA) for 80 min. To quantify the total protein, the resulting blots were incubated in red-colored Ponceau S for 5 min. Non-specific binding was blocked overnight with 5% non-fat dry milk in Tris base saline with 0.05% Tween 20 (TBS-T).

The next day, blots were washed three times in TBS-T (the washing solution was used between each step) and incubated with the following HRP-conjugated primary antibodies: anti-VDAC1 (sc-390996, 1:500; Santa Cruz Biotechnology, Dallas, TX, USA) for 1 h; anti-GRP75 (sc-133137, 1:500; Santa Cruz Biotechnology, Dallas, TX, USA) and anti-NCX1 (ab17795, 1:1000; Abcam, Cambridge, UK) for 2 h; anti-PLN (sc-393990, 1:500; Santa Cruz Biotechnology, Dallas, TX, USA) for 3 h; anti-MCU (CST14997, 1:1000; Cell Signaling Technology, Danvers, MA, USA), anti-SERCA2a (sc-53010, 1:500; Santa Cruz Biotechnology, Dallas, TX, USA), anti-RyR (ab219798, 1:500; Abcam, Cambridge, UK), and anti-IP3R (ab108517, 1:500; Abcam, Cambridge, UK) overnight.

Then, membranes were incubated with the appropriate secondary antibodies for 1 h: rat anti-mouse IgG-HRP (ab131368, 1:10,000; Abcam, Cambridge, UK) for VDAC1, GRP75, and PLN; goat anti-mouse IgG-HRP (sc-2005, 1:1000; Santa Cruz Biotechnology, Dallas, TX, USA) for SERCA2a; goat anti-rabbit IgG-HRP (A9169, 1:20,000; Sigma-Aldrich, Munich, Germany) for NCX1, MCU, RyR, and IP3R. Finally, the immunoreactive proteins were visualized after 5 min exposition to Clarity™ Western ECL chemiluminescent substrate in the dark using a ChemiDoc™ XRS Imaging System and quantified in the Image Lab™ program (both Bio-Rad Laboratories, Hercules, CA, USA). Results were normalized to total protein and expressed as relative protein levels in arbitrary units (AU).

### 2.7. Data Analysis

Data were explored and analyzed in R (https://www.R-project.org/, accessed on 15 December 2021) ver. 4.0.5., with the aid of several libraries, most notably, lme4 and DHARMa. To make the research reproducible, the analyses were written in an R notebook. The data were summarized by the mean, median, standard deviation, and inter-quartile range. A spaghetti plot was used to visualize the longitudinal data. A quantile–quantile plot with a 95% confidence interval constructed by the bootstrap method was used to assess the normality of the continuous variables. Repeated-measures two-way ANOVA was fitted to the data using the linear mixed model, which in the Wilkinson Rogers notation can be expressed as response~treatment*age + (1|id). The deviance residuals were used for the diagnostic analysis of the model (normality of noise, homoskedasticity, outliers). The marginal means were used to derive the estimates of the mean and its 95% confidence interval and pairwise post hoc comparisons with the Tukey adjustment of *p*-values. Marginal and conditional R2 were used to measure the effect size. An interaction plot was used to visualize the marginal means. Results are presented as mean ± standard deviation (SD) and findings with a *p*-value below 0.05 were considered statistically significant.

## 3. Results

### 3.1. Effect of IR on Contractile Function Parameters during Aging

**Figure 2:**
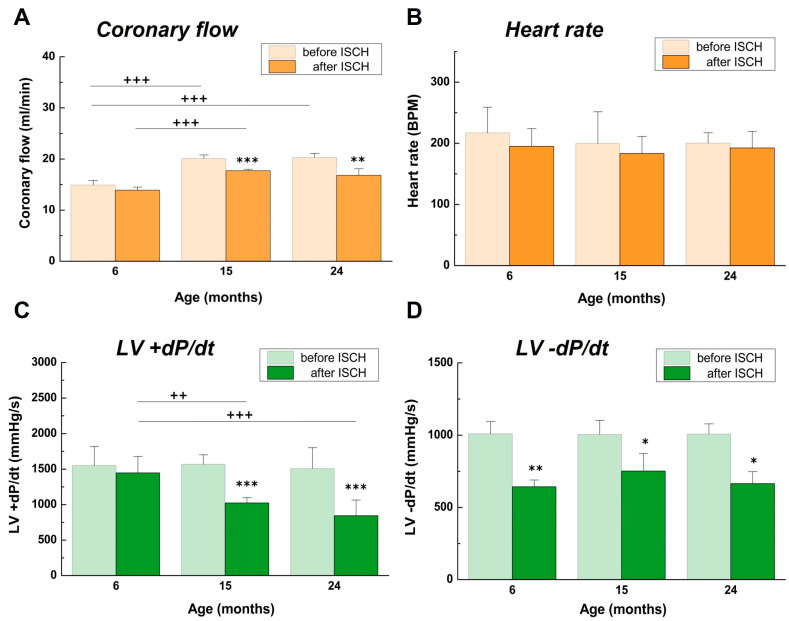
Effect of IR on contractile function parameters during aging. Individual parameters are presented as (**A**) coronary flow, (**B**) heart rate, (**C**) LV +dP/dt (maximum rate of pressure development), and (**D**) LV −dP/dt (maximum rate of relaxation). Values are expressed as mean ± SD of 5 hearts per each experimental group, * *p* < 0.05, ** *p* < 0.01, *** *p* < 0.001, significantly different when compared to the preischemic value, or ^++^ *p* < 0.01, ^+++^ *p* < 0.001, significantly different when compared between age groups.

The effect of IR on parameters of contractile function in aging hearts is summarized in Figure 1. The sensitivity of the hearts to IR changed with age when the coronary flow decreased, most significantly in 15-month-old and 24-month-old hearts (Figure 2A). Heart rate was not affected by IR as well as age itself (Figure 2B). The maximum rate of pressure development (LV +dP/dt) was maintained in adult post-ischemic hearts and throughout whole aging before ISCH, but age-related deprivation occurred after IR (Figure 2C).

The most significant changes were shown in the senescent group, with a 43.9% decrease (*** *p* < 0.001) from 1506.4 mmHg/s to 843.7 mmHg/s after IR. The initial value of the maximum rate of relaxation (LV −dP/dt) was maintained during aging. However, the response to IR was different among the individual age groups, with the most sensitive 6-month-old hearts reaching only 62.6% of pre-ischemic value (** *p* < 0.01) in comparison to the 15- and 24-month-old groups (Figure 2D). Altogether, contractile parameters were affected by IR, but they differ in the ability to restore their functions with age.

**Figure 3:**
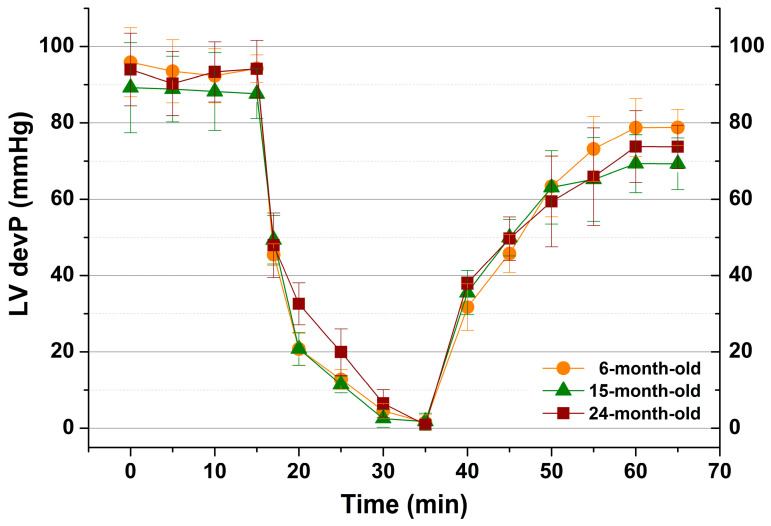
Recovery of LV devP after myocardial IR during aging. Values are expressed as mean ± SD of 5 hearts per each experimental group. *p* > 0.05 represents tendency but no significant changes between age groups. LV devP is Left Ventricular developed Pressure.

The preischemic values of LV devP were similar in all age groups. The recovery after 20 min of ischemia was slightly deteriorated in aged and senescent hearts but changes were not significant. At the end of reperfusion, recovery reached 83.6% for adults, 79.1% for old, and 78.3% for senescent hearts (Figure 3).

### 3.2. Ca^2+^-ATPase Activity in Aging Hearts after IR

**Figure 4:**
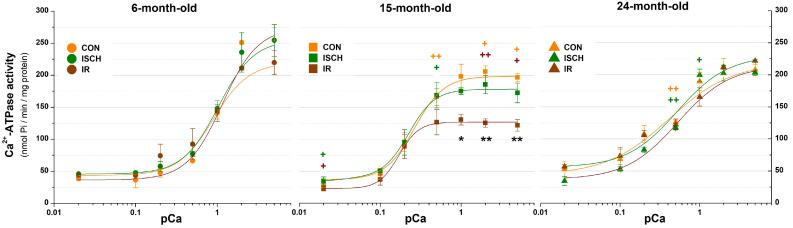
Calcium dependence of Ca^2+^-ATPase activity during aging in CON, ISCH, and IR groups. The nonlinear least-squares method with Hill fitting was used for the graphical interpretation of Ca^2+^ dependence. Values are expressed as mean ± SD of 5 hearts per each experimental group. * *p* < 0.05, ** *p* < 0.01, significantly different when compared to the CON, or ^+^ *p* < 0.05, ^++^ *p* < 0.01, significantly different when compared between age groups.

Aging itself as well as acute myocardial IR influenced to some extent Ca^2+^-ATPase (SERCA2a) activity in sarcoplasmic reticulum. We recorded very stable Ca^2+^-dependent activity profile changes in 24-month-old hearts in comparison to 6- and 15-month-old hearts exposed to IR. At higher free [Ca^2+^] concentrations (1.0–5.0 μM), 6-month-old hearts were able to reach 83.2% of the maximum velocity measured in control-perfused hearts. On the other side, the Ca^2+^-binding ability of 15-month-old hearts was highly affected in response to IR reaching only 63.9% of control. This points toward impaired Ca^2+^-sequestering capacity of old cardiomyocytes (Figure 4), since the greater portion of Ca^2+^-ATPase was inhibited at low free Ca^2+^ concentrations up to 0.1 μM after IR.

As an independent risk factor, aging had an impact on Ca^2+^-ATPase activity in control-perfused hearts, with a decrease in maximum velocity from 264.1 ± 7.5 in 6-month-old to 218.1 ± 2.1 nmol P_i_/min/mg protein in 24-month-old hearts. However, Ca^2+^-ATPase activity did not change at low 0.02–0.5 μM free [Ca^2+^].

### 3.3. Impact of Myocardial IR on SERCA2a and PLN Contents during Aging

**Figure 5:**
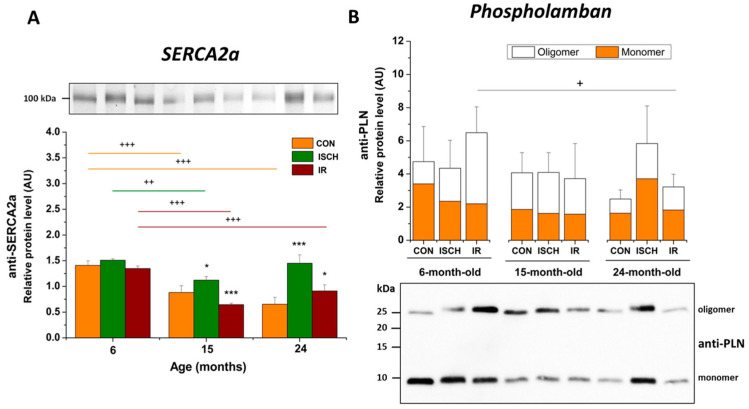
The relative protein levels of (**A**) SERCA2a and (**B**) PLN after IR in aged hearts. The total PLN amount is presented as the sum of PLN monomers and oligomers. Values are expressed as mean ± SD of 3 hearts per each experimental group. * *p* < 0.05, *** *p* < 0.001, significantly different when compared to the CON, or ^+^ *p* < 0.05, ^++^ *p* < 0.01, ^+++^ *p* < 0.001, significantly different when compared between age groups. SERCA2a (SR/ER Ca^2+^-ATPase), PLN (phospholamban).

The aging process gradually decreased SERCA2a protein levels in cardiomyocytes, but each age group had a different response to ISCH/IR. Unlike activity, the protein level of SERCA2a did not change in the youngest group of 6 months, while, in the group of 15-month-old group, the decrease in SERCA2a positively correlated with its activity reduction after IR. The basal level for SERCA2a was altered during aging with a significant 53.5% decrease (*** *p* < 0.001) in senescent individuals compared to adults (Figure 5A).

The most critical for myocardial contraction is SERCA2a coexpression with phospholamban (PLN), which exists in a dynamic equilibrium of oligomeric states ranging from pentamer to monomer. In our study, the formation of PLN oligomers and monomers was age-dependent in response to ISCH or IR. Total PLN elevation accelerates cytotoxic effects, causing inhibition of Ca^2+^-ATPase in 6-month-old hearts subjected to IR (Figure 5B). Contrary to this, a relatively stable PLN was detected in 15-month-old cardiomyocytes. In senescent hearts, changes in PLN levels mirrored the response of SERCA2a to ischemia and reperfusion.

### 3.4. Impact of Myocardial IR on the Level of Other Ca^2+^-Regulating Proteins during Aging

**Figure 6:**
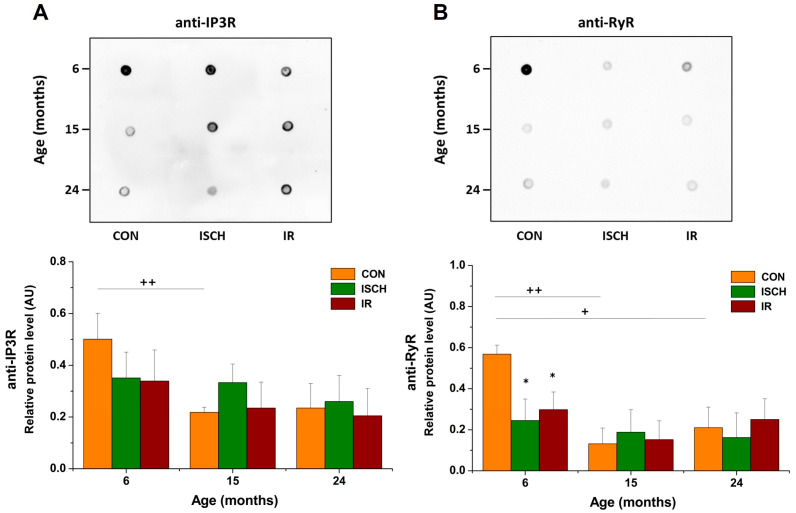
Representative dot blots with the relative protein levels of IP3R (**A**) and RyR (**B**) after IR in aged hearts. Values are expressed as mean ± SD of 3 hearts per each experimental group. * *p* < 0.05, significantly different when compared to the CON, or ^+^ *p* < 0.05, ^++^ *p* < 0.01, significantly different when compared between age groups. IP3R (inositol 1,4,5-trisphosphate receptor), RyR (ryanodine receptor).

Heart homogenates were used to visualize various Ca^2+^-regulating proteins in aging cardiomyocytes subjected to IR, with a specific focus on the communication between ER/SR and mitochondria. Ca^2+^ release channels, IP3R and RyR, had the same trend of expression changes with a decrease in RyR level by 43.6% after IR in the 6-month-old group, but with age, there was significant suppression of both proteins (Figure 6A,B).

**Figure 7:**
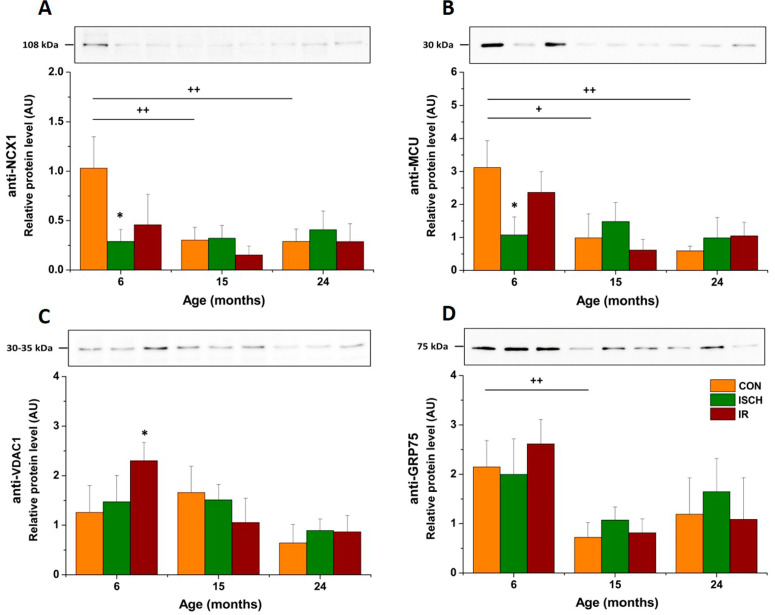
Representative Western blots with the relative protein levels of NCX1 (**A**), MCU (**B**), VDAC1 (**C**), and GRP75 (**D**) after IR in aged hearts. Values are expressed as mean ± SD of 3 hearts per each experimental group. * *p* < 0.05, significantly different when compared to the CON, or ^+^ *p* < 0.05, ^++^ *p* < 0.01, significantly different when compared between age groups. NCX1 (Na^+^/Ca^2+^ exchanger), MCU (mitochondrial Ca^2+^ uniporter), VDAC1 (voltage-dependent anion channel 1), GRP75 (glucose-regulated protein 75).

During ischemia, a decrease in pH can inhibit proteins or stimulate their downregulation (* *p* < 0.05), such as a 3.6-fold decrease in the Na^+^/Ca^2+^ exchanger NCX1 (Figure 7A) and a 2.9-fold reduction in mitochondrial Ca^2+^ uniporter MCU in the 6-month-old hearts (Figure 7B). Upon reperfusion, NCX1 as well as MCU did not change.

VDAC1 upregulation by 82.6% (Figure 7C) on the outer mitochondrial membrane can help to maintain Ca^2+^ transmission into the mitochondria with the support of higher GRP75 (Figure 7D). Adult hearts were highly sensitive to myocardial IR in comparison to 15- and 24-month-olds, but the aging process itself caused, to a different extent, significant deprivation of Ca^2+^-handling protein levels.

## 4. Discussion

During aging, deterioration in cardiac structure and function [45] elevates the susceptibility of the heart to ischemia and myocardial infarction [9,46]. These complex morphological and functional changes involve a change in shape and loss of cardiomyocytes [16], increased inflammation, hypertrophy, fibrosis [47], and left ventricular remodeling with compensatory wall thickening [48], all contributing to the contractile efficiency of the heart that declines with age. While patients older than 65 years old have usually confirmed diastolic dysfunction [49], changes in systolic function are not consistent and provide conflicting data across studies. In our experimental model, the sensitivity of hearts to IR changed with age. The decline in coronary flow was most significant in a post-ischemic group of 15-month-old rats. Such changes have a direct impact on cardiac contractility, which falls suddenly in post-ischemia infarction and might induce heart failure or even cardiomyocyte death [10]. We did not observe significant age-related differences in the baseline contractile function of isolated rat hearts. These results agree with studies on rodents and human myocardium, where the contractility was not substantially altered with age [3,50,51]. A study using a similar Langendorff model showed that aging does not modify the time to onset and peak contracture [4]. While similar mild IR-induced diastolic (LV −dP/dt) dysfunction was present in all groups, significant deterioration of LV +dP/dt was observed in aged and senescent hearts. Published studies investigating the effect of 30 min ischemia showed that the first 5 min of reperfusion are critical for recovery of contractile function [52,53,54]. The recovery of LV devP was not significantly different among the groups, but restoration of contractility was slightly lower in the aged groups, mainly in the 15-month-old group. Similar findings were observed by [4]. No age-dependent differences were found by [5,6]. Since the restoration of contractility seems to be slightly affected by aging, it may affect the intervention time point and therapeutic effect of young and elderly patients.

This phenomenon should cause corresponding non-constant changes in contractility and related changes at the cellular level, such as age-related stress intolerance, mitochondrial dysfunction, free radical accumulation, and Ca^2+^-handling impairment [55,56]. In cardiomyocytes, intracellular Ca^2+^ homeostasis is maintained by Ca^2+^ influx and SR–Ca^2+^ storage [29]. Depolarization induces a transient rise in intracellular Ca^2+^ through the L-type Ca^2+^ channel and subsequently triggers Ca^2+^ release through the ryanodine receptors and Ca^2+^ release channels as IP3Rs [31] to accomplish contraction. During relaxation, intracellular Ca^2+^ is extruded from the cytosol via the Na^+^/Ca^2+^ exchanger NCX1 and sequestered into the sarcoplasmic reticulum [41] by the activity of SERCA2a [29,57]. Unlike SERCA2a (high affinity/low capacity), NCX1 (low affinity/high capacity) responds rapidly to changes in intracellular Ca^2+^ and ensures ion exchange 3Na^+^/Ca^2+^ [58]. However, the direction depends on actual intracellular concentration of Na^+^, Ca^2+^, and membrane potential changes. The age-dependent decline in NCX1 protein levels was detected in both 15- and 24-month-old groups compared to adult hearts. Post-ischemic reduction in NCX1 in adult hearts affects its functionality during reperfusion, where it may operate in reverse mode, causing Ca^2+^ overload and subsequent worsening of cardiac damage. While mice with NCX1 deletion were resistant to cardiac IR damage due to the inhibition of Ca^2+^ overload [59], another study showed that high expression of NCX1 suppresses the development of systolic and diastolic dysfunction in patients with heart failure [60]. However, excessive NCX1 activity is associated with reduced SERCA2a efficiency during diastolic Ca^2+^ reuptake [61], causing Ca^2+^ transient impairment [34] and loss of sarcoplasmic reticulum Ca^2+^ stores.

Age had a significant impact on basal Ca^2+^-ATPase activity in perfused rat hearts at higher 1.0–5.0 μM free [Ca^2+^]. Moreover, age-dependent reduction in SERCA2a expression (53.5% decrease in senescent individuals) might be associated with prolonged SR–Ca^2+^ transients [31,32]. On the contrary, elevated SERCA2a was detected on the atrioventricular junction of 24-month-old rat hearts [35], which highlights possible location-specific compensatory mechanisms in elderly hearts. The most critical for myocardial contraction is SERCA2a coexpression with phospholamban, which is expressed abundantly in ventricles [62] and exists in a dynamic equilibrium of oligomeric states ranging from pentamer to monomer. We observed a gradual decline in the total PLN by 28.5% in 15-month-old and by 56.3% in 24-month-old rats along with the aging-dependent decrease in SERCA2a. When comparing PLN monomers to total PLN, we found that the 6- and 24-month-old groups had higher content of monomers. The total and monomeric PLN mirrored the response of SERCA2a overexpression after IR in 24-month-old hearts, thus contributing to stable Ca^2+^-transporting capacity and overall ATPase activity. Since Ca^2+^ influx into the SR is an ATP-dependent process (~15% of cardiac energy usage), overexpression of SERCA2a could increase energy requirements in 24-month-old rats, but lower ATP reserve may contribute to contractile dysfunction.

Previous studies have shown that the inhibitory activity of PLN is attributed to its monomeric form, as seen in PLN mutagenesis experiments [63] or reciprocal SERCA2a–PLN interaction studies, where SERCA2a increase was accompanied by a decrease in oligomerization of PLN [64]. These results suggest that SERCA2a sequesters monomeric PLN and disruption of PLN–SERCA2a interactions can improve cardiac systolic dysfunction [65]. We have shown IR-induced reduction in SERCA2a content and significant inhibition of Ca^2+^-ATPase activity at low free Ca^2+^ concentrations up to 0.1 μM in 15-month-old hearts, with the ratio of PLN to SERCA2a contents higher in comparison to 6- or 24-month-old hearts. Since a comparative study (6- vs. 26-month-old animals) reported that only 40% of SERCA2a is normally regulated by PLN [66], the elevated PLN-to-SERCA2a ratio may suggest that a larger part of SERCA2a is inhibited at lower Ca^2+^ concentrations in 15-month-old. This points towards impaired Ca^2+^-sequestering capacity of aged cardiomyocytes subjected to IR. Ca^2+^-ATPase activity in 6-month-old post-ischemic hearts was affected at higher (2–5 μM) free Ca^2+^ with unchanged SERCA2a protein levels. The overall PLN, as well as the ratio of PLN to SERCA2a, massively increases after IR in the 6-month-old group, which can slow down Ca^2+^ reuptake by SERCA2a. These changes are mostly seen in senescent 24-month-old rats [29,33]. Moreover, previous studies have shown that high PLN expression has cytotoxic effects on SERCA2a [67], leaving a lot of questions regarding the structure or mutual interactions (e.g., mono-/oligomer exchange, oligomer–SERCA dynamics) unanswered.

Sarcoplasmic reticulum-derived Ca^2+^ is essential for ECC, and RyR2 regulates Ca^2+^ release from SR to the cytosol [31]. It was proposed that the downregulation of RyR2 before ischemia can reduce Ca^2+^ overload [68]. On the contrary, during IR the structure of RyR2 is damaged, which triggers Ca^2+^ leakage from SR and subsequent Ca^2+^ overload [69]. We have seen an age-dependent reduction in RyR2 content in control groups and IR-induced downregulation of RyR2 by 32.3% in 6-month-old hearts. Moreover, the inhibition of another Ca^2+^-releasing protein, IP3R, during IR suggested that its cardioprotective effect could be relevant for therapeutic strategies aimed at modulating the interaction of mitochondria and SR [70]. The proximity of VDAC1 and Ca^2+^-regulatory proteins allows Ca^2+^ transfer to mitochondria [71], where VDAC1 in a closed state has a high affinity for cations such as Ca^2+^ [72]. In the present study, VDAC1 content did not change with age, but a significant increase in expression was observed after IR in 6-month-old hearts. This may increase the leakage of Ca^2+^ from the sarcoplasmic reticulum and contribute to mitochondrial Ca^2+^ overload. Recently, the VDAC1–citrate synthase co-immunostaining study confirmed an increase in VDAC1 per unchanged mitochondrial density [73]. The presence of the cardiotoxic isoproterenol stimulated VDAC1 levels in H9c2 cardiomyoblast cells [74] with the promotion of mitochondria-mediated cell death, which could reflect an increase in the level of VDAC1. This was confirmed by another study, in which VDAC1 was downregulated by antioxidant resveratrol without the presence of cardiomyocyte apoptosis, while VDAC1 overexpression abolished its cardioprotective effect [73].

GRP75 as a modulator of the IP3R–VDAC1 complex increases the efficiency of Ca^2+^ uptake by mitochondria [75]. We have shown a potentially protective decrease in GRP75 during aging, especially in 15-month-old cardiomyocytes. This is supported by studies where the complete depletion of GRP75 exerted protection against stress with maintained mitochondrial homeostasis [76]. The inner mitochondrial membrane Ca^2+^ uniporter multi-protein complex mediates Ca^2+^ uptake into the matrix and subsequently activates the synthesis of ATP [38]. Throughout the years, studies published a lot of conflicting results. In the present study, we observed a decrease in basal MCU content with age, but the ability of the hearts to contract was not impaired. Cardiac-specific deficiency of MCU augmented fatty acid oxidation [77] and functional energy reserve [78]. Although several animal models have been developed, results remain contradictory, with the underlying mechanisms largely unknown.

The limitation of this study is that experiments were performed only on male rats. Therefore, we cannot exclude that, due to hormonal effects, the tolerance to ischemic injury in female rats will differ from that observed in the present study. The presented experimental model is suitable to monitor acute ischemic injury that leads to complete recovery in time. For this reason, the phenomenon of stunning may lead to a delay in post-ischemic recovery of cardiac function, and prolonged periods of reperfusion will also be appropriate for testing.

## 5. Conclusions

In conclusion, our study suggests that aging is associated with a significant decrease in the abundance and function of Ca^2+^-handling proteins. However, the IR-induced damage was not increased during aging. These findings do not support the frequent view on the role of aging in the susceptibility to heart ischemia–reperfusion injury. Nevertheless, they suggest that SERCA2a, RyR, and other Ca^2+^-handling proteins as well as proteins involved in SR–mitochondria cross-talk may serve as suitable target molecules for therapy of age-associated alterations in heart function.

## Data Availability

All data related to this work can be made available upon request to the corresponding authors.
